# Periodontal and Dental Pulp Cell-Derived Small Extracellular Vesicles: A Review of the Current Status

**DOI:** 10.3390/nano11071858

**Published:** 2021-07-19

**Authors:** Shu Hua, Peter Mark Bartold, Karan Gulati, Corey Stephen Moran, Sašo Ivanovski, Pingping Han

**Affiliations:** 1 Epigenetics Nanodiagnostic and Therapeutic Group, Center for Orofacial Regeneration, Rehabilitation and Reconstruction (COR3), School of Dentistry, Faculty of Health and Behavioural Sciences, The University of Queensland, Brisbane, QLD 4006, Australia; s.hua@uq.net.au; 2 School of Dentistry, The University of Queensland, Brisbane, QLD 4006, Australia; mark.bartold@adelaide.edu.au (P.M.B.); k.gulati@uq.edu.au (K.G.); corey.moran@uq.edu.au (C.S.M.)

**Keywords:** extracellular vesicles, exosomes, nanomedicine, regeneration, cell-free therapy

## Abstract

Extracellular vesicles (EVs) are membrane-bound lipid particles that are secreted by all cell types and function as cell-to-cell communicators through their cargos of protein, nucleic acid, lipids, and metabolites, which are derived from their parent cells. There is limited information on the isolation and the emerging therapeutic role of periodontal and dental pulp cell-derived small EVs (sEVs, <200 nm, or exosome). In this review, we discuss the biogenesis of three EV subtypes (sEVs, microvesicles and apoptotic bodies) and the emerging role of sEVs from periodontal ligament (stem) cells, gingival fibroblasts (or gingival mesenchymal stem cells) and dental pulp cells, and their therapeutic potential *in vitro* and *in vivo*. A review of the relevant methodology found that precipitation-based kits and ultracentrifugation are the two most common methods to isolate periodontal (dental pulp) cell sEVs. Periodontal (and pulp) cell sEVs range in size, from 40 nm to 2 μm, due to a lack of standardized isolation protocols. Nevertheless, our review found that these EVs possess anti-inflammatory, osteo/odontogenic, angiogenic and immunomodulatory functions *in vitro* and *in vivo*, via reported EV cargos of EV–miRNAs, EV–circRNAs, EV–mRNAs and EV–lncRNAs. This review highlights the considerable therapeutic potential of periodontal and dental pulp cell-derived sEVs in various regenerative applications.

## 1. Introduction

Extracellular vesicles (EVs) are membrane-bound bilayered lipid particles that are secreted from both prokaryotic and eukaryotic cells, carrying a cargo of biological molecules (i.e., protein, nucleic acid, lipids and metabolites) from their parent cells [1]. Initially, EVs were considered ‘cellular dust’, generated by cellular metabolism, until their biological role in the mineralization of bone was recognized [2,3]. A principal role of EVs is as an intercellular communicator of biological information into a recipient cell. This interaction can trigger signaling cascades and modulate cell behavior [4]. The biological function of EVs is defined by the parent cells from which they originate. EVs are involved in almost all cellular interactions, especially tumor metastasis, tissue homeostasis, and inflammatory regulation [4,5]. Due to their constituent biological molecules, EVs hold great promise as a therapeutic delivery system in regenerative medicine.

**Figure 1:**
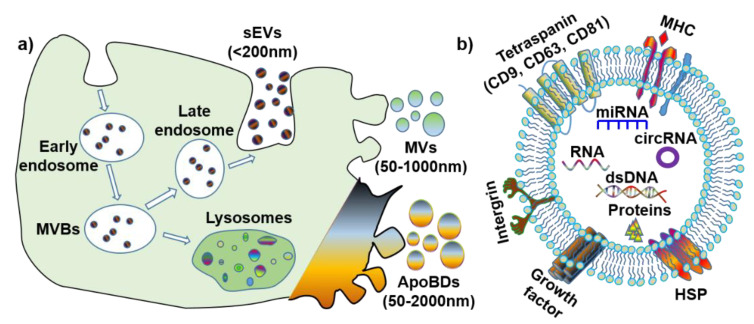
The biogenesis and contents of extracellular vesicles (EVs). (**a**) Biogenesis and size of three EV subtypes. (**b**) Common surface markers and cargos of EVs. sEVs: small extracellular vesicles; MVs: microvesicles; ApoBDs: apoptotic bodies; MVBs: multi-vesicular bodies; MHC: major histocompatibility complex; HSP: heat-shock protein; dsDNA: double-stranded DNA; miRNA: microRNA; circRNA: circular RNA.

The definition, terminology and subtypes of EVs are still being debated. The International Society of Extracellular Vesicles (ISEV) recommends a division of EV subtypes based on their size: medium/large EV (>150 nm) and small EV (<150 nm) [6]. However, considering the discrepancies in the published literature, for simplification purposes, this review will define EV subtypes based on both their size and biogenesis (Figure 1a): small extracellular vesicles (also known as exosomes) (sEVs, <200 nm), microvesicles (MVs, 50–1000 nm) and apoptotic bodies (ApoBDs, 50–2000 nm). Furthermore, it is noteworthy that all EVs have various membrane proteins (e.g., tetraspanin, MHC, and HSP) and components (e.g., dsDNA, RNA, microRNA, circular RNA [7], and proteins) (Figure 1b).

### 1.1. Small EV (Exosomes)

Small EVs (sEV), or exosomes, originate from endosomes, and are biological nanoparticles that are smaller than 200 nm [5]. Further, sEVs are produced through an endocytic pathway, and their particle size is partially overlaid with that of microvesicles and apoptotic bodies. The biogenesis process for sEV is unique, whereby the endosomal network is the source of sEV that produce, classify, distribute and define the proper destination of the secreted sEV [2,8]. Endosome production can be categorized into the following three subtypes, according to each stage of development: early endosomes, late endosomes, and recycling endosomes. Early endosomes are formed by inward budding of the cell membrane, before a second inward budding of the endosomal membrane that results in the formation of late endosomes—intraluminal vesicles (ILVs). Late endosomes containing IVLs are named multi-vesicular bodies (MVBs), and the MVBs either fuse with lysosomes to degrade or follow the endocytic pathway for sEV generation. Once fusion with the plasma membrane is completed, the small membrane-enclosed vesicles are released into the extracellular matrix.

The biogenesis of sEV is affected by the following two main pathways that can induce multi-vesicular bodies (MVBs) generation: the endosomal sorting complex, required for the transport (ESCRT)-dependent pathway and ESCRT-independent pathway [9]. For the ESCRT-dependent pathway, ESCRT I and ESCRT II mediate the invagination of the late endosomal membrane, and ESCRT III will be recruited to the invaginated membrane sites. The cargo proteins are then deubiquitinated, and this stimulates the departure of the vesicle and the formation of MVBs. In the ESCRT-independent pathway, the neutral sphigomylinase2 (nSMase2) takes sphingolipids as substrates and converts sphingolipids to ceramide at the endosomal membrane. Following this, the microdomain is prepared for merging into a larger structure, which accelerates the endosomal budding and biogenesis of MVBs [10]. Moreover, sEVs that are produced by these different pathways possess different biomarkers, except CD63, which is the most common biomarker for all sEVs [11,12]. With respect to the ESCRT-dependent pathway, if endocytosis is mediated by Ras-related protein 27A/B (RAB27A/B), TSG101 is a biomarker of sEV. If the endocytosis is mediated by phospholipase D2 (PLD2) and RAB7, through the ESCRT-independent pathway, the biomarkers of sEV are alix, syntenin, and syndecan. As for the RAB11/35-mediated ESCRT-independent pathway, CD81, Wnt and proteolipid protein (PLP) are the preferred biomarkers.

The function of sEV in intercellular communication is determined by the interconnection between sEV surface proteins and receptors on the recipient cells that subsequently activates a variety of signaling pathways [5]. Further, sEVs arising from different cell types have different cargos that dictate and direct different biological effects. The sEVs are highly abundant in biofluids [13,14,15], and they have been demonstrated to be associated with immune response, viral pathogenicity, osteogenesis, odontogenesis, neuroprotection, angiogenesis, and anti-tumor functions [16]. For example, oral cancer cell-derived sEVs create a mechanism that can promote tumor progression by modifying vesicular contents and establishing a distant premetastatic niche with molecules that favor cancer cell proliferation, migration, invasion, metastasis, angiogenesis, and even drug resistance [17]. Evidence that sEVs play an important role in cell differentiation suggests that sEVs may have a potential role in tissue regeneration.

### 1.2. Microvesicles

Microvesicles (MVs) are membrane vesicles of different sizes, surrounded by a lipid layer of membrane, and they range in size from 50 nm to 1 μm. Microvesicles are generated by the outward budding of the plasma membrane, and are abundant in tissues/cells and biofluids [18]. The contents of MVs are similar to that of sEV. The MV components of note include CD40, selectins, integrins, cytoskeletal proteins, and cholesterol [19].

The biogenesis of MV involves the contraction of cytoskeletal proteins and phospholipid redistribution, contributing to a dynamic interplay in the plasma membrane and the resultant formation of microvesicles. Within the plasma membrane, the aminophospholipid translocase regulates phospholipid distribution, transferring phospholipids from one leaflet to another. Once phosphatidylserine (PS) is translocated to the leaflet of the outer membrane, the outward blebbing of the membrane and microvesicle formation is initiated. The interaction between actin and myosin causes the cytoskeletal structure contraction, which mediates membrane budding [20].

MVs have been reported to maintain tissue homeostasis during tissue regeneration, angiogenesis, anti-tumor effects, and in pathologies such as tumorigenesis, chronic inflammation, and atherosclerosis [19]. MVs that are produced by blood cells (e.g., neutrophils, macrophages, and platelets) are involved in the pro-coagulatory response [21]. MVs can be both pro-inflammatory and anti-inflammatory; this is determined by the induction or stimulation that is received by their parent cells. MVs that are produced by tumor cells enhance invasiveness and accelerate cancer progression, as well as strengthen the drug resistance of tumor cells [22]. This indicates that MVs are potential therapeutic agents for tissue regeneration; however, the function of MVs in periodontal tissue healing and regeneration requires further investigation.

### 1.3. Apoptotic Bodies

Apoptotic bodies (ApoBDs) are produced by cells undergoing apoptosis, and vary in size from 50 nm to 2 µm [23,24]. ApoBDs result from the formation of subcellular fragments when an apoptotic cell disassembles. They are comprised of molecular components from living cells and provide a rich molecular pool for recipient cells. However, ApoBDs are engulfed by macrophages and digested by phagolysosomes shortly after they are released [25]. ApoBDs and apoptosis are not related to an inflammatory reaction, the constituents in dying cells and ApoBDs are not released automatically to the environment, and anti-inflammatory cytokines are not generated during engulfing. ApoBDs have phosphatidylserine (PS) on their surfaces, to attract engulfing cells, and are considered to be specific biomarkers for ApoBDs [26]. Autoimmune diseases may be associated with defects in the clearance of ApoBDs. ApoBDs may stimulate the formation of thrombus and improve anti-cancer immunity.

Increasing evidence suggests that ApoBDs have important immune regulatory roles, in autoimmunity, cancer, and infection [24], as well as promoting osteogenesis [27]. For example, ApoBDs that are derived from mature osteoclasts can induce osteoblast differentiation by activating the protein kinase B/phosphoinositide 3-kinases (PI3K/AKT) pathway [27]. However, knowledge of their function and role is still limited and more studies are required in this field.

## 2. The Source and Characteristics of Periodontal (Dental Pulp) Cells

**Figure 2:**
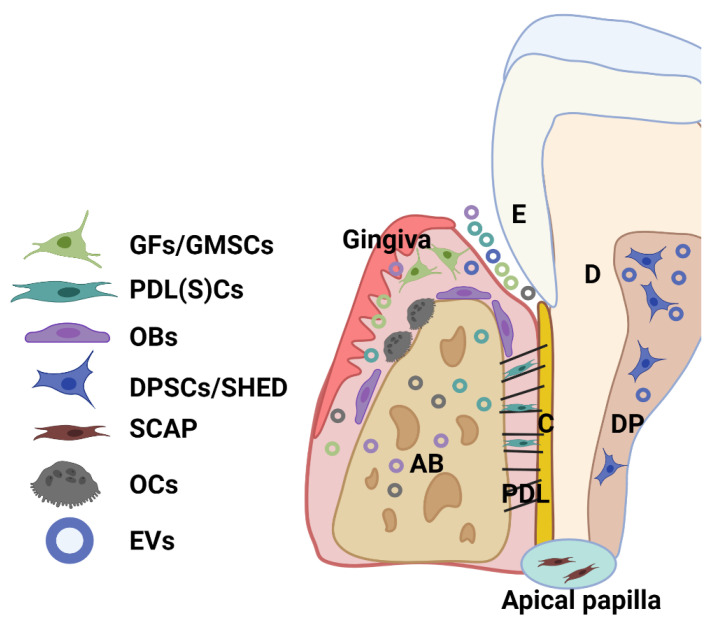
Schematic showing the main cell population and cell products (EVs) within a healthy periodontium. Various cells reside in the periodontium, such as periodontal ligament (stem) cells (PDLSCs), fibroblasts (GFs) and stem cells (GMSCs) from the gingiva, osteoblasts (OBs), osteoclasts (OCs), and various immune cells. AB: alveolar bone; C: cementum; D: dentin; DP: dental pulp; PDL: periodontal ligament; SHED: dental pulp cells from human exfoliated deciduous teeth (SHED); SCAP: cells from periodontal apical papilla tissues (SCAP).

Dental tissue-derived (or stem) cells have remarkable characteristics for therapeutic application, being easily accessible and a rich source of stem cells with a well-known regenerative capacity. A great variety of multipotent adult or postnatal stem cells can be retrieved from dental tissues, especially from periodontal tissue and dental pulp from extracted permanent teeth (dental pulp stem cells—DPSCs) and exfoliated deciduous teeth (SHED). A healthy periodontium consists of soft (periodontal ligament-PDL and gingiva) and hard (alveolar bone and cementum) tissue, and cells residing within the healthy periodontal tissues include periodontal ligament (stem) cells (PDLSCs), PDL and gingival fibroblasts (PDLF, GFs), or gingival stem cells (GMSCs), osteoblasts (OBs), osteoclasts (OCs), and various immune cells (Figure 2) [28,29]. Moreover, stem cells can be obtained from dental apical papilla tissues (SCAP) and dental follicles (DFSCs, or DFCs) of the developing tooth [28,29]. Importantly, EV that is derived from these cells can be detected within periodontal tissues and biofluid (i.e., gingival crevicular fluid) (Figure 2).

Dental mesenchymal stem cells originate from the neural crest ectomesenchyme and reside in stromal niches (perivasculature and peripheral nerve-associated glia cells). The current consensus holds that both perivascular cells [30] and glia cells [31] are responsible for dental MSCs origin, as revealed in mouse experiments [31]. Much like bone-marrow-derived MSCs that originate from mesoderm [32], dental stem cells express MSCs markers and exhibit multipotent linage regeneration (i.e., osteogenic, chondrogenic, neurogenic) and immunomodulatory capabilities. These properties make these cells suitable candidates for therapeutic application (reviewed by Chalisserry et al. in [33]) in neurological disorders, angiogenesis, dentin-pulp regeneration and periodontal regeneration. PDLSCs, GFs, DPSCs, SHED, and DFSCs have been demonstrated to promote multiple-tissue regeneration, both *in vitro* and *in vivo* [34,35,36,37,38,39,40]. However, cell therapy has several challenges, including high cost, insufficient cell number, and associated regulatory barriers. On the other hand, a cell-free approach, centered around cell products (i.e., EVs derived from these cells), has been proposed, and there is an emerging focus on cell-derived EVs as potential therapeutic agents to promote periodontal regeneration. The utilization of sEVs for dental tissue regeneration is emerging as a viable cell-free treatment option, with ‘proof of concept’ studies reported using bone marrow or adipose MSC-derived sEVs (reviewed in [41,42,43]); yet, periodontal or dental pulp cell sources are likely to uniquely reflect the functional complexity of the periodontium and oral cavity.

The following sections will summarize the current methods for cell-derived sEV isolation and characterization, with particular emphasis on sEVs from periodontal and dental pulp cells.

## 3. Cell-Derived sEV Isolation Methods

### 3.1. General Concepts

Although sEVs have been studied for decades, there is still no standardized protocol for their isolation. Despite the presence of recommended guidelines for EV isolation and characterization, such as the Minimal Information for Studies of Extracellular Vesicles 2014 (MISEV2014) and MISEV 2018, these guidelines are not always followed.

Prior to the isolation of sEV, sequential centrifugation is commonly used to remove cell debris and large EVs, as follows:

- Step 1: the cell conditional media (CM) is harvested and centrifuged at 300–400× *g* to remove cells, and the supernatant (SN) is collected;
- Step 2: the SN collected in step 1 is centrifuged at 2000–3000× *g* to remove cells debris and apoptotic bodies. The SN is collected from this step;
- Step 3: SN from step 2 is centrifuged at 10,000–20,000× *g* to remove the aggregates of biopolymers, microvesicles, and the other structures with a buoyant density higher than sEVs. The SN is collected from this step;
- Step 4: then, the following isolation methods are used to enrich the sEVs: ultracentrifugation, sucrose gradient centrifugation, size exclusion chromatography, precipitation-based isolation, immunoaffinity chromatography, and ultrafiltration.

Given the growing interest in EVs, technical standardization is critical, as many different methodologies have been utilized for isolation and analysis. The influence of these various techniques on the downstream composition and functionality of EV cargos remains unclear; accordingly, the ISEV position papers [6,44] have raised the need to define ‘good practices’ and ultimately archive standardization. However, many researchers are not following these four steps, due to a lack of standardized protocols. Here, our review briefly introduces each isolation method, and discusses its merits and disadvantages (listed in Table 1).

### 3.2. Ultracentrifuge

Ultracentrifugation is the gold-standard method for isolating sEV, as the equipment is relatively easy to access and the methodology is technically straightforward. The method involves an ultracentrifugation step at 100,000×–200,000× *g* to pellet sEV [45]. However, ultracentrifugation has disadvantages, in that it leads to a low recovery rate of sEV, it is time consuming (1.5–10 h), contains non-vesicular macromolecule contamination, and results in EV aggregation.

### 3.3. Floatation-Related Methods (Sucrose Gradient Centrifugation)

Floatation-related methods distribute molecules based on the buoyant density, and the protein aggregates and sEV can be sufficiently separated. Differential gradient centrifugation (usually takes 250 min—1 day) takes advantage of buoyant density to fractionate EVs using sucrose or idoxanol gradients [45]. The sEVs can be separated by the discontinuous gradient sucrose solution, with each layer containing the desired size of EV. Other chemical reagents (i.e., iodixanol) can also be utilized instead of sucrose, for continuous EV harvest with no layers. Non-vesicular protein contaminants are distributed at a reduced level within this method, resulting in less protein contamination. However, sucrose gradient centrifugation cannot separate large particles that have a similar sedimentation rate.

### 3.4. Size Exclusion Chromatography (SEC)

SEC can be used to isolate small sEV, based on the size of the molecules, where large particles pass through the gel earlier than the small-sized molecules. The small-sized particles are trapped in the tiny pores on the surface of the gel, while the larger molecules can bypass the gel or receive less interference from the gel [46]. This technique has been well established with commercialized SEC columns, including qEV (iZON Science), Exo-spin™ SEC columns (Cell Guidance Systems Ltd.) and Pure-EVs SEC columns (HandaBioMed, Lonza). SEC has been proposed as an effective alternative method for pure sEV isolation, with a key advantage being its time efficiency (~30 min, including 10 mL of column washing with PBS). However, the similarly sized sEV and microvesicles cannot be separated by SEC.

### 3.5. Precipitation-Based Isolation (Sodium Acetate, PEG, Protamine)

Precipitation-based isolation has the following two mechanisms: polymeric precipitation and neutralizing charges [47]. In polymeric precipitation, a soluble polymer, usually polyethylene glycol (PEG), is mixed with EV samples and the mixture is incubated overnight, and EVs are sedimented by low-speed centrifugation at 1500 g. PEG precipitation enables a simple process for a large number of samples. Commercial kits, such as ExoQuick (System Biosciences), total exosome isolation reagent (Invitrogen), EXO-Prep (HansaBioMed), exosome purification kit (Norgen Biotek), and miRCURY exosome isolation kit (Exiqon), are based on this principle. For the other precipitation method, all EVs possess negative charges, so positively charged molecules (i.e., sodium acetate and protamine) are chosen for the precipitation. This method is popular due to its straightforward protocol; however, these precipitation methods lead to low sEV purity due to co-precipitation of the components from CM or biofluids, such as protein, DNA and RNA, and hence further purification is required.

### 3.6. Immunoaffinity Chromatography

The monoclonal antibodies (mAbs) against specific sEV surface proteins (i.e., CD 9) are fixed on the column, to capture a specific sEV population [48]. Once the CM passes through the column, the EVs, which express certain exosomal markers on their membrane, will be captured by the mAbs. This method leads to a very pure EV population, but low yield and scalability. This is attributed to the fact that this step needs to be repeated several times to ensure the mAbs can capture sufficient EVs (~240 min).

### 3.7. Ultrafiltration

Semi-permeable membranes (ranging from 3 kDa to 100 kDa) are adapted for sEV fractionation within filtration-based isolation; the membrane function is determined by its pore size. However, sEVs cannot be fractionated according to their biogenesis or biomarkers, but it is normally used to concentrate sEVs. It is still an efficient way to eliminate the minimal sample volume (~130 min) with a simple procedure, and has been proven to yield higher recovery of sEVs than ultracentrifugation [49]. However, ultrafiltration might lead to low EV protein, but a rather higher concentration of non-EV proteins (i.e., albumin).

### 3.8. Current Isolation Challenge

As mentioned previously, the current challenges of sEV isolation include time-consuming procedures, impurities, insufficient EV yield, and low scalability [50]. Although many researchers have investigated combinations of these isolation methods, an urgent demand has arisen to investigate high-yielding and time-effective isolation protocols. Currently, there is no optimal sEV isolation method; however, a combination of ultracentrifugation, SEC, and ultrafiltration has been used for the pure sEV population, which is a critical factor for downstream therapeutic applications.

## 4. sEV Isolation and Characterization Methods for Periodontal (and Dental Pulp) Cells

**Figure 3:**
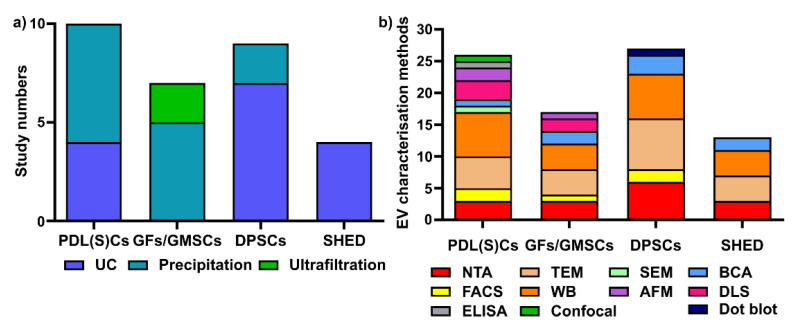
Various sEV isolation methods (**a**) and characterization methods (**b**) are used for periodontal (dental pulp) cells. UC: ultracentrifugation; NTA: nanoparticle tracking analysis; TEM: transmission electron microscopy; WB: Western blot; SEM: scanning electron microscopy; AFM: atomic force microscopy; BCA: bicinchoninic acid assay; DLS: dynamic light scattering; ELISA: enzyme-linked immunosorbent assay; confocal: confocal microscopy.

To date, there are no standardized protocols for sEV isolation and characterization. From the 33 studies that are reported in this review, we have summarized periodontal (dental pulp) cell-derived sEV isolation and characterisation methods [51,52,53,54,55,56,57,58,59,60,61,62,63,64,65,66,67,68,69,70,71,72,73,74,75,76,77,78,79,80,81,82,83]. Various isolation methods have been used for periodontal (dental pulp) cells sEVs, including ultracentrifugation (UC), precipitation-based methods, and ultrafiltration (Figure 3a). Regarding EV characterization, the latest MISEV 2018 guidelines [6] suggest that all EV researchers should characterize sEV from at least three different aspects, such as EV particle numbers, EV morphology, and EV-enriched protein markers. However, most of the current studies did not follow the MISEV guidelines, and this requires additional attention for all EV research.

Different sEV isolation methods have been utilized for various cells (Figure 3a), with precipitation and ultracentrifugation methods being the two most commonly used techniques. In PDL(S)C-derived sEV isolation (10 studies), the precipitation-based method (i.e., a commercial ExoQuick kit) is the most commonly used (*n* = 6, 60%), followed by ultracentrifugation (*n* = 4, 40%). Among six studies in GFs/GMSC-derived sEVs, the precipitation-based method (*n* = 4, 66.7%) and ultrafiltration (*n* = 2, 33.3%) were used for GFs/GMMSCs–sEV isolation. Regarding DPSC-derived sEV, most researchers selected ultracentrifugation (*n* = 7, 77.8%), with one study using the precipitation-based method (*n* = 2, 22.2%). For SHED–sEVs, all of the studies (*n* = 4, 100%) used the ultracentrifugation method to isolate sEVs from SHED.

Concerning EV characterisation [6], NTA and DLS are common methods to quantify EV particle number, size, and distribution; TEM, SEM, and AFM can be used for EV morphology and size; BCA is for EV protein quantification; and WB is to determine EV-enriched protein markers. We have summarized the various EV characterisation methods for periodontal cell-derived sEVs (Figure 3b). For PDL(S)Cs—sEV (10 studies included in this review), WB is the most commonly used characterization method (*n* = 7 studies), followed by TEM (*n* = 5), NTA (*n* = 3), AFM (*n* = 2), flow cytometry (*n* = 2), SEM (*n* = 1), BCA assay (*n* = 1), ELISA (*n* = 1), and confocal microscopy (*n* = 1). In GFs/GMMSCs–sEVs, WB (*n* = 4) was utilized to detect CD9, CD63, and TSG101, as well as TEM (*n* = 4), NTA (*n* = 3), DLS (*n* = 2), BCA assay (*n* = 2), AFM (*n* = 1), and FACS (*n* = 1). The characterisation of DPSC–EVs are mostly performed using WB (*n* = 7), TEM (*n* = 8), NTA (*n* = 6), BCA assay (*n* = 3), FACS (*n* = 2), and dot blot (*n* = 1). For the characterization of SHED–sEVs (4 studies), TEM and WB (*n* = 4) are most commonly applied; NTA (*n* = 3) and BCA (*n* = 2) were also used for OBs–sEVs.

In summary, ultracentrifugation and precipitation-based methods are the two most common methods used for periodontal (dental pulp) cells sEV isolation. WB, TEM and NTA are the most common methods for periodontal (dental pulp) cell-derived sEVs characterisation.

## 5. The Function of sEVs Derived from Periodontal (Dental Pulp) Cells

Current studies mainly focus on small EV biogenesis and function in the periodontal regeneration field; thus, this review summarizes 33 studies [51,52,53,54,55,56,57,58,59,60,61,62,63,64,65,66,67,68,69,70,71,72,73,74,75,76,77,78,79,80,81,82,83] on periodontal cell-, gingival cell- and dental pulp (DPSCs and SHED) cell-derived sEV isolation, characterization, and their therapeutic role in tissue regeneration. Most of this research has focused on the function of sEVs in cell differentiation, and 11 studies investigated the cargos of sEVs (i.e., miRNA [52,53,61,63,64,72,75,81], circRNA [51,71], lncRNA [51], and EV-mRNA [67,72]) during this process.

### 5.1. Periodontal Ligament Fibroblasts or Stem Cells (hPDL(S)Cs)–sEV

A total of 10 studies investigated sEVs derived from human PDL (stem) cells or fibroblasts [51,52,53,54,55,56,57,58,59,60], and are summarized in Table 2. Eight studies isolated sEV from hPDLSCs [51,52,53,55,56,58,59,60], with one study each using sEVs from a human PDL fibroblast (hPDLFs) cell line [54] and hPDLCs [57]. Three of these studies investigated hPDLCs sEV function *in vivo*, using animal models [56,59,60].

According to the latest MISEV 2018 guidelines [6], it is critical to consider several factors influencing the collection of EV, including characteristics of primary cell source (donor health status, age, gender), primary cell passage number, confluence at harvest, culture volume, media change frequency, CM harvesting conditions, as well as all culture media composition and preparation details. Thus, our review includes detailed donor information for primary cells (if mentioned), the cell culture condition prior to CM collection, and detailed sEV isolation protocols. This will allow future EV researchers to select appropriate protocols for CM harvesting and sEV isolation.

Donor age was disclosed in only two studies (18–30 [51] and 18–21 years old [55]), while there is no clear information in the other studies [52,53,54,56,57,58,59,60]. The cells at passages 2–3 were used in five studies [51,52,53,56,59], passage 3–7 in one study [54], with no passage information provided in the remaining studies [55,57,58,60]. Since fetal calf serum (FBS or FCS) contains a large amount of EV, it is crucial to state how cells are cultured before CM harvesting. Currently, either EV-depleted FBS or FBS starvation is used before CM harvest for PDLCs–sEV isolation; from the 10 studies that were reviewed, 5 did not state how the cells were cultured before CM collection [53,56,58,59,60], 4 studies used EV-depleted FBS [52,54,55,57], and one study used FBS starvation [51]. While it is of considerable importance to clearly articulate the cell source, passage number, and CM harvest condition, this is something that is currently under-reported in many studies.

The following three aspects of hDPL(S)Cs–sEV analysis were evaluated in the 10 studies that have been included in this review: (1) EV size, (2) EV content (protein, RNA, etc.), and (3) EV function in cell differentiation *in vitro* and *in vivo*. Regarding the size of hPDL(S)Cs–sEV, three studies did not characterize the sEV size [52,53,60]. There is a large deviation for the reported EV size: <200 nm in five studies [51,54,55,57,58], two populations (90 ± 20 nm and 1200 ± 400 nm) in one study [59], and 100–710 nm in one study [56]. It is noted that two studies engineered the hPDLCs–EV using polyethyleneimine (PEI, yielding PEI–EV) [56,59]. The following two factors may contribute to this deviation: the EV isolation method (UC or precipitation methods), and the EV size characterization methods (TEM, or DLS, or NTA). We will define EV size <200 nm as sEV, and unclear EV size as EV.

Regarding the EV content, it seems that hPDLCs–EV contain miRNAs [52,53] and circular RNAs [51] that may alter the recipient cells functions. RNA sequencing of hPDLSCs–EV (where EV size was unclear) revealed that hPDLSCs–EV contains 955 non-coding transcripts, with five representative miRNAs, including MIR24-2, MIR142, MIR296, MIR335, and MIR490 [53]. The hDPLSCs–EV–miR-17-5p can regulate the angiogenesis of human umbilical vein endothelial cells (HUVECs) during inflammatory stimulation by TNF-α [52]. Furthermore, circular RNA and long non-coding RNAs (lncRNAs) were also found in the sEV from hPDLSCs, after five and seven days of osteogenic differentiation, with 69–557 circRNAs and 2907–11,581 lncRNAs detected by RNA sequencing. Compared with the sEV from hPDLSCs before osteoinduction, 3 sEV–circRNAs and 2 sEV–lncRNAs were upregulated, while 39 sEV–circRNAs and 5 sEV–lncRNAs were downregulated after 5 and 7 days of osteoinduction. RT-qPCR validation showed that three sEV–circRNAs (*hsa_circ_0087960*, *hsa_circ_0000437*, and *hsa_circ_0000448*) were upregulated after osteogenic differentiation, while one was downregulated (*hsa_circ_0000448*). However, three selected lncRNAs (*small nucleolar RNA host gene5*—*SNHG5*, *LOC100130992,* and *ATP6VB1-AS1*) showed no difference between the groups [51].

There is increasing evidence demonstrating that hPDL(S)Cs–EV can modulate *in vitro* angiogenesis (in HUVECs [52]), osteogenesis (in MG-63 OBs [54] and hPDLSCs [55,56,59]), anti-inflammation (in LPS-treated hPDLSCs [55,60] and J774.1 macrophages [57]), and immunoregulation (induced M1 polarization in THP-1 cells [58]) via modulating the TGF-beta pathway, MAPK pathway, mTOR pathway and FoxO signaling pathways [51], and PI3K/Akt signaling [55] and NF-kB signaling pathways [57]. The *in vivo* function of hPDL(S)Cs–EV was explored, either in rat calvaria defect [56,59] or intravenous administration in mouse multiple sclerosis disease [60] models. Pizzicannel-la et al. [56] created a calvarial defect, with a diameter of 4 mm and a height of 0.25 mm in male Wistar rats (300–350 g; *n* = 4 for each group). The hPDLSCs–EV and hPDLSCs–PEI–EV were loaded on collagen membranes and transplanted into the rat calvaria defect for 6 weeks, leading to enhanced bone and vascularization compared to the no-EV groups, with the PEI–EV group inducing better osteogenesis and vascularization compared to the EV group. Diomede et al. [59] revealed similar results, showing that hPDLSCs–PEI–EV leads to increased blood vessel formation after 6 weeks of the transplantation of hPDLSCs–EV- and hPDLSCs–PEI–EV-loaded collagen membranes into a rat calvarial defect. Rajan et al. [60] established a mouse model of MS disease, and intravenous administration of hPDLSCs–EVs decreased apoptosis and inflammation in the diseased mice.

In summary, the size of hPDL(S)Cs–EV ranges from 20 nm to 1600 nm when using different EV isolation methods, with under-reporting of sufficient detail about the cell source and cell culture conditions before CM collection. The hPDL(S)Cs–EV contains miRNAs, circRNAs, and lncRNAs, and they modulate the angiogenesis, osteogenesis, and inflammation of recipient cells, through TGF-β, MAPK, mTOR and FoxO pathways [51], and PI3K/Akt [55] and NF-kB signaling pathway [57]. However, none of the three *in vivo* studies [56,59,60] used either a periodontal defect or a periodontitis animal model.

### 5.2. Human Gingival Fibroblasts (hGFs)–sEV

Table 3 summarizes seven studies [61,62,63,64,65,66,67]) of EV from fibroblasts (hGFs [62,63] or MSCs (hGMSCs [61,64,65,66,67]) from human gingiva tissues. There are two studies that investigated the *in vivo* role of hGMSCs–EV using animal models [66,67]. The cells from either 20-to-40-year-old donors [66] or unclear age human donors [62,63,64,65,67] were used at passage 2 [64], passage 4–6 [62], <6 passage [66], or unclear [63,65,67]. EV-depleted FBS [61,63,66], FBS starvation [65], and unclear cell culture conditions [62,64,67] were applied in the studies before CM collection for EV isolation. The size of hGFs/hGMSCs–EV varied from different studies, as follows: <200 nm [61,62,63,66], 50–500 nm [65], unclear size [64], and a combination of two populations (93 ± 24 nm and 1200 ± 400 nm) [67]. Engineered hGMSCs–PEI–EV had the following two populations: 250 ± 50 nm and 3600 ± 500 nm [67].

RNA sequencing data from Silvestro et al. showed that hGMSCs–EV comprises 15,380 genes (for interleukins, TGF-β, BMPs, GDFs, Wnt, VEGF, FGF, and neurotrophins), and 1155 non-coding RNA (lncRNAs and miRNAs—miR1302, miR451, miR24, miR219 and miR194) [64]. The miRNA microarray data from Nako et al. [61] showed that 655 universal differentially expressed miRNAs were found in Exo-TNF compared to Exo-Ctrl, particularly miR-1260b (ranked in the top three of the most highly upregulated miRNAs, by using TNF- α preconditioning). RNA sequencing from Diomede et al. [67] demonstrated that 31 ossification genes were enhanced in hGMSCs–PEI–EV compared to hGMSCs-EV through the TGF-β signaling pathway.

The *in vitro* functional assays showed that hGFs/hGMSCs–sEV facilitates cell proliferation (in hGFs [62] and Schwann cells line [66]), anti-osteoclastogenic [61] and osteogenic differentiation (in hBMSCs [63] and hGMSCs [67]), as well as an anti-carcinogenesis effect (in human pancreatic carcinoma and squamous carcinoma cells [65]). This may be mediated by an miR-1260b/Wnt 5A/RANKL pathway [61], miR-23a/CXCL12 axis [63], interleukins, TGF-β, BMPs, GDFs, Wnt, VEGF, FGF, and neurotrophins [64], and TGF-β signaling [67].

In their *in vivo* investigations, Nakao et al. [61] created a ligature-induced periodontitis mice model, and locally injected hGMSCs–sEV or TNF-α-preconditioned GMSC-derived exosomes (hGMSCs–sEV–TNF) into the palatal gingiva of the ligated second maxillary molar. One week post-injection, both the interventions significantly reduced periodontal bone loss compared to the PBS control group, while hGMSCs–sEV–TNF further reduced the distance from the cementoenamel junction to the alveolar bone crest (CEJ–ABC) and the number of tartrate-resistant acid phosphatase (TRAP)-positive osteoclasts, indicating an anti-osteoclastic property for hGMSCs–sEV [61]. Moreover, Mao et al. [66] transplanted hGMSCs–sEV-loaded gelfoam sheets into the crush-injury sites of sciatic nerves in C57BL/6J mice, and the EV group had comparable beneficial effects on the functional recovery of the injured sciatic nerves of mice compared to the hGMSCs group. Further, hGMSCs–sEV enhanced the expression of neuronal and Schwann cell markers (β-tubulin III and S100 calcium-binding protein B—S100B) at one-month post-injury, compared with hGMSCs controls, suggesting that hGMSCs–sEV can promote neuron regeneration *in vivo*. Diomede et al. [67] loaded hGMSCs–EV or hGMSCs–PEI–EV into 3D-printed PLA scaffolds with/without hGMSCs, and transplanted them into rat calvaria defects for 6 weeks. Both the hGMSCs–EV and hGMSCs–PEI–EV groups enhanced bone and blood vessel formation, yet hGMSCs–PEI–EV performed better than the EV group.

In summary, the EV diameter from hGFs/hGSMCs is different among studies, ranging from 50 nm to 1600 nm. EV–mRNAs and EV–miRNAs [61,64,67] may contribute to their *in vitro* and *in vivo* function in cell proliferation [62,66], and reduce bone resorption [61], osteogenic differentiation [63,67] and nerve regeneration [66]. More *in vivo* studies are required in order to explore the function of EV from gingival tissue-derived cells.

### 5.3. Human Dental Pulp Cells (hDPSCs)–sEV

Table 4 summarizes nine studies investigating EV from human primary DPSCs [68,69,70,71,72,73,74,75,76], with three of these including *in vivo* models [69,72,76]. Cells were isolated from donors who were 24–41 years old [69], 16–25 years old [70], 20 years old [71], 19–28 years old [73], 22–36 years old [75], or an unclear donor age [68,72,74,76]. The cells at passage 2 [71], passage 3–6 [70], passage <4 [76], passage 3–7 [75], passage 3–5 [73], or unclear passage number [68,69,72,74] were used in these studies. Prior to CM collection, the cells were cultured in EV-depleted FBS [68,70,71,72,73] or FBS starvation [69,74,75,76]. The mode size of hPDSCs–sEV was smaller than <200 nm in most studies [68,69,70,71,72,75], with one study reporting 50–400 nm [72], 80–400 nm [73], and 30–250 nm [74], and unclear EV size [76].

The hPDSCs–EV modulates angiogenesis in endothelial cells [69], migration/proliferation (in hBMMSCs [70], Schwann cells (SCs) [73], and CD4+ T cells [74]), osteogenic differentiation in hDPSCs [71], anti-inflammation (in DPSCs [72] and CD4+ T cells [74]), and odontogenic differentiation of Schwann cells (SCs) [73], hDPSCs [75,76], and hMSCs [76]. This may be regulated through hDPSCs–sEV–circPAR1 binding with hsa-miR-31 [71], hDPSCs–sEV–miR-1246 [72] and hDPSCs–sEV–miR-27a-5p [75]. RNA sequencing data from Hu et al. [75] demonstrated that 7 increased sEV–miRNAs and 21 decreased sEV–miRNAs were found in odontogenic differentiated hDPSCs–sEV, and these miRNAs are associated with the TGFβ1/smads signaling pathway. The authors concluded that sEV–miR-27a-5p can modulate odontogenic differentiation via the TGFβ1/smads signaling pathway, by downregulating latent-transforming growth factor beta-binding protein 1 (LTBP1).

With respect to *in vivo* studies, Zhou et al. [69] created a full-thickness excisional skin wound-healing model in male C57BL/6 mice (8 weeks old), and then subcutaneously injected hDPSCs–sEV (200 μg in 100 μL) from healthy or periodontitis patients derived hDPSCs–sEV (200 μg in 100 μL PBS) for 4, 9, and 14 days. Both the sEV groups promoted the wound healing process and vascularization compared to the PBS control group, while hDPSCs–sEV from the periodontitis patients increased the wound closure rate and the number of newly formed microvessels, with more CD31- and VEGF-positive cells compared to the sEV from a healthy patient. Shen et al. [72] established a ligation-induced periodontitis model in 6–8-week-old male C57BL/6J mice, and a chitosan hydrogel (CS) loaded with 50 μg of hDPSCs–sEV (hPSDCs–sEV–CS group) was locally injected after ligature removal, with a local injection of PBS or hPSDCs–sEV used as the controls. The results showed that the hDPSCs–sEV–CS group led to increased bone formation, a thick layer of epithelial layers, less inflammatory cells, and a lower amount of TRAP-positive osteoclasts, at 10 days post-treatment. Furthermore, hPSDCs–sEV–CS treatment significantly reduced pro-inflammatory cytokines (IL-23, IL-1α, TNF-α, IL-12, IL-1β, IL-27, and IL-17), and NF-κB p65 and p38 MAPK signaling, in periodontal tissues compared with other groups. RNA sequencing analysis of the periodontium showed that 7351 differentially expressed genes (DEGs) were found between the hDPSCs–sEV–CS and CS groups. GO term enrichment analysis of the top 200 DEGs demonstrated that they are associated with chemotaxis pathways and the immune response, which were downregulated in the hDPSCs–sEV–CS group. Most importantly, hDPSCs–sEV–CS induced macrophages converting from a proinflammatory phenotype to an anti-inflammatory phenotype in the periodontium of periodontitis mice, with more CD206+ anti-inflammatory macrophages and significantly decreased CD86+ in pro-inflammatory macrophages [72]. This indicates that hDPSCs–sEV can promote bone formation, epithelium re-growth, and reduce inflammation in a periodontitis mice model. Furthermore, Huang et al. [76] loaded hDPSCs–EV into clinical-grade type I collagen membranes, and then placed them on a human tooth root slice (3–4 mm in thickness), before subcutaneously transplanting into athymic nude mice for 2 weeks. They resulted in enhanced dental-pulp-like tissues, with increased odontogenic proteins (dentin matrix acidic phosphoprotein 1—DMP1, and dentin phosphophoryn—DSPP) and endothelial cell marker protein (von Willebrand factor—vWF).

To summarize, hDPSCs–EV, ranging from 30 nm to 400 nm among nine studies, and containing circRNA [71], miRNAs [72,75], and mRNAs [72], may modulate angiogenesis [69], migration/proliferation [70,73,74], osteogenic differentiation [71], anti-inflammation [72,74] and odontogenic differentiation [73,75,76] in recipient cells. Among the three *in vivo* studies, the skin wound-healing model [69], periodontitis disease model [72], and subcutaneous transplantation [76] were employed, and the results showed that hPDSCs–EV can promote angiogenesis, osteogenesis, dentin-pulp regeneration, and reduce inflammation and osteoclastic activity. Further *in vivo* studies are required to validate the function of hDPSCs–EV.

### 5.4. SHED/SCAP/DFCs–sEVs

Table 5 summarizes seven investigations (five *in vivo* studies) examining sEVs from dental cells, including SCAP [77,78], SHED [79,80,81,82], and DFCs [83]. Cells were isolated from 5–8-year-old donors [81], 12–15 years old [77,78], 13–19 years old [83], or unknown age [79,80,82], at passage 3–4 [79], 4–7 [80], 4 [81], 3–6 [82], 5 [83], or unknown [77,78]. EV-depleted FBS [77,79,81,82] and FBS-starvation [78,80,83] were applied for CM collection.

The size of SHED/SCAP/DFCs–sEVs was smaller than 200 nm in all the studies; these sEVs promote angiogenesis in HUVECs [77,82], anti-inflammation in mBMSCs [80] and chondrocytes [81], osteogenesis in PDLCs [79], mBMSCs [80] and rBMSCs [82], and dentinogenesis in BMMSCs [78,83] *in vitro*, by the Cdc42 pathway [77], Wnt/β-catenin and BMP/Smad signaling pathways [79], miR-100–5p/mTOR pathway [81], and AMPK pathway [82].

The function of SCAP–sEVs was investigated *in vivo* on gingival soft tissue [77] and dentin-pulp regeneration [78]. Liu et al. [77] created full-thickness circular gingival wounds in C57BL/6J mice, using a biopsy punch (soft tissue defects with a diameter of 2.0 mm). Following this, 40 μg of SCAP–sEVs, SCAP–siCdc42–sEVs, or PBS, was injected submucosally into the palates of the wounds sites. Seven days post-injection, SCAP–sEVs promoted palatal gingival tissue regeneration by enhancing vascularization in the early phase [77]. Zhuang et al. [78] loaded 50 μg/mL SCAP–sEVs and 4 × 10^5^ BMMSCs with gelatin sponge onto a dentin slice, before subcutaneously transplanting them into immunodeficient mice. Significant dentin-pulp regeneration was observed 12 weeks post-transplantation in the SCAP–sEVs group compared to the PBS control group.

The action of SHED–sEVs on periodontitis disease and periodontal defect *in vivo* has been investigated in a mouse [80] and rat model [82], respectively. Wei et al. [80] locally injected 20 µg of SHED–sEVs into buccal and lingual sides of the first molar once per week, over 2 weeks, in ligature-induced periodontitis mice. After 2 weeks, SHED–sEVs reduced bone loss, with a decreased CEJ–ABC distance compared to the controls. Moreover, Wu et al. [82] generated a periodontal defect (4 × 2 × 1.5 mm^3^) in their rat model, at the buccal alveolar bone of the first-to-third mandibular molars. SHED–sEVs were loaded into a β-TCP scaffold before placing them into the periodontal defect for four weeks, resulting in enhanced neovascularization and new bone formation compared to the β-TCP/PBS scaffold.

In their study, Shi et al. [83] injected gelatin hydrogels (100 µL), loaded with LPS–DFCs–sEVs (sEVs derived from LPS-treated DFCs) or DFCs–sEVs, into the periodontal pocket of the right maxillary second molar in a ligature-induced periodontitis rat model. The intervention was once a week for up to 8 weeks, and resulted in significantly reduced alveolar bone loss and TRAP-positive osteoclasts, as well as enhanced well-oriented PDL fibers in the LPS–DFCs–sEVs group.

In summary, SHED/SCAP/DFCs–sEVs are smaller than 200 nm, and those containing miR-100–5p [81] may modulate angiogenesis [77,82], inflammation [80,81], osteogenesis [79,80,82], and dentinogenesis [78,83] *in vitro*. More importantly, five *in vivo* studies showed that SHED/SCAP/DFCs–sEVs can promote angiogenesis [77,82], dentin-pulp complex [78], alveolar bone [82], and well-organized PDL fiber formation [82]. It is noted that two studies utilized a ligature-induced periodontitis disease model [80,83] and one study used a periodontal defect model [82]. More studies are needed to further validate the *in vivo* functional role of SHED/SCAP/DFCs–sEVs.

## 6. Summary and Discussion

Periodontal cells (PDLCs/SCAP and GFs/GMSCs) and dental pulp (DPSCs/SHED)-derived EVs can play an important role in augmenting the function of recipient cells, such as proliferation and osteo/odontogenic differentiation, as well as anti-inflammation and anti-cancer properties [51,52,53,54,55,56,57,58,59,60,61,62,63,64,65,66,67,68,69,70,71,72,73,74,75,76,77,78,79,80,81,82,83]. In particular, one study of GMSCs–sEVs [61] and DPSCs–sEVs [72], two studies of SHED–sEVs [80,82], and one study of DFCs–sEVs [83] can promote alveolar bone, vasculature and well-organized PDL fibers regeneration, and reduced inflammation in a periodontitis animal model or a periodontal defect model. As such, these EVs may serve as potential ‘cell-free’ therapeutics to facilitate periodontal regeneration; however, more *in vivo* studies are required to confirm this concept.

As stated in the latest MISEV 2018 guidelines [6], it is critical to clearly describe the primary cell source (i.e., donor age, health status, gender), primary cell passage number, cell culture conditions (using either EV-depleted FBS or FBS starvation before CM collection), and detailed EV isolation and characterization protocols. Among 33 studies in our review, only two studies used human or mouse cell lines [54,57], and 31 studies isolated EV from primary cells, with only 12 out of 31 studies stating a clear age range for the human or mouse donors [51,55,66,69,70,71,73,75,77,78,81,83], and 13 out of 31 studies were unclear about cell passage numbers [55,57,58,60,63,65,67,68,69,72,74,77,78]. Since FBS is largely EV contaminated, EV-depleted FBS or FBS starvation should be used for cell culture before CM collection. EV-depleted FBS was used in 12 studies [61,63,66,68,70,71,72,73,77,79,81,82], FBS starvation in 8 studies [65,69,74,75,76,78,80,83], and unclear cell culture conditions in 11 studies. Although all the studies used the two most common sEV (or exosome) isolation methods (precipitation and ultracentrifugation), the EV size in these studies (excluding studies with no EV characterization) is not consistent, with 22 studies generating <200 nm sEVs [51,54,55,57,58,61,62,63,66,68,69,70,71,72,75,77,78,79,80,81,82,83]. This may be attributed to the different CM collection, EV isolation and characterization methods among the studies. Thus, appropriate methods should be chosen to prepare CM, and isolate and characterize cell-derived EV according to the MISEV guidelines. Our review has defined <200nm EV as sEV (small EV) and unclear size or >200 nm as simply EV.

Among 33 studies, 12 studies performed *in vivo* research to investigate the EV function of hPDLCs–sEV [56,59,60], hGMSCs–EV [66,67], hDPSCs–EV [69,72,76], SCAP–sEVs [77,78], SHED–sEVs [80,82] and DFCs–sEVs [83]. Furthermore, three studies engineered the EV using polyethyleneimine (PEI), yielding PEI–EV [56,59,67], and all three studies reported that the PEI–EV group enhanced *in vivo* osteo/odontogenic and angiogenic properties compared to the EV group. Animal studies employed either defect or disease models, such as calvaria defects [56,59,67], nerve injury model [66,67], skin wound-healing model [69], subcutaneous transplantation [76], and multiple sclerosis [60], ligation-induced periodontitis [69,72,80,83] and a periodontal defect [82].

EVs were administrated either by loading into biomaterials, such as collagen membrane [56,59,76], gelfoam sheets [66], gelatin sponge [78] and 3D-printed PLA scaffold [67], or via intravenous administration [60], subcutaneous injection [69], local injection [61,72,80,83], or submucosal injection [77]. More pre-clinical models (i.e., periodontal defects or periodontitis disease models) and EV delivery systems need to be investigated to explore the potential of periodontal cell-derived EV in the regeneration of anatomically complex tissues, such as the periodontium.

All of the above factors are critical for a successful therapeutic outcome; thus, it is of great importance to follow the relevant guidelines and consider the above-discussed variables with more comparisons between different parameters.

## 7. Conclusions and Future Perspectives

**Figure 4:**
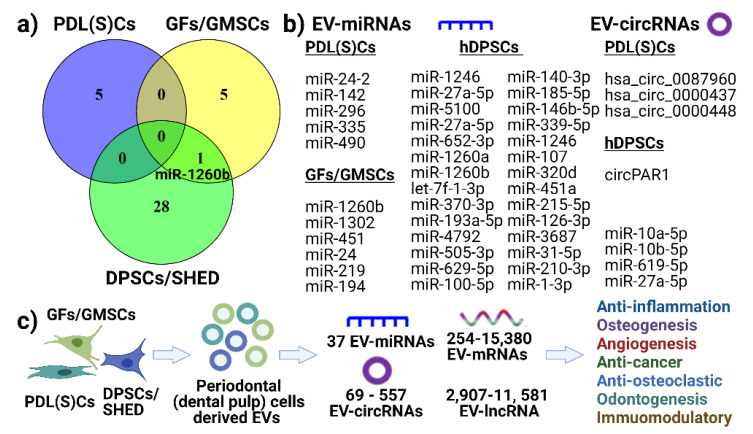
Summary of EV–miRNAs, circular RNAs (**a**,**b**) and proposed (**c**) function of periodontal cell-derived EV on recipient cells function. (**a**) Venn diagram showing no common EV–miRNAs found from PDL(S)Cs, GFs/GMSCs and DPSCs. (**b**) Listed EV–miRNAs and EV–circRNAs. (**c**) Proposed mechanism of how periodontal cell-derived EVs modulate inflammation, angiogenesis, osteo/odontogenesis via EV cargos, such as miRNA, mRNAs, lncRNAs, and circRNAs.

This review demonstrates that sEV can be isolated from periodontal and pulp cells, with 11 studies investigated the EV cargos, including sEV–miRNA [52,53,61,63,64,72,75,81], EV–circRNA [51,71], EV–lncRNA [51] and EV–mRNA [67,72]. We summarize the common EV–miRNA and EV–circRNA within periodontal (or dental pulp) cells (Figure 4a,b). From the included studies, except for one common EV–miRNA (miR-1260b) between DPSCs/SHED and GFs/GMSCs, there appears to be no common EV–miRNA detected between these cell types (shown in Venn diagram, Figure 4a). We also listed reported EV–miRNAs and EV–circRNAs from PDL(S)Cs–EV and hDPSCs-–EV (Figure 4b). However, this needs further confirmation with more studies. Furthermore, 38 EV–miRNAs, 69–557 EV–circRNAs, 254–15,380 EV–mRNAs and 2907–11,581 EV–lncRNAs were reported for EV from periodontal (dental pulp) cells by RNA sequencing analysis. We have outlined that these EVs possess anti-inflammation, osteo/odontogenesis, anti-osteoclastogenesis, angiogenesis and immunomodulatory functions *in vitro* and *in vivo*. Thus, we propose that periodontal cell-derived EVs can modulate the cell function via EV cargos (Figure 4c). However, more studies for periodontal cell-derived EVs are required to further confirm this concept.

Given that cell source, CM collection, and EV isolation and characterization are critical in obtaining pure EV populations, future studies should take these factors into account and follow the latest MISEV guidelines. Researchers should consider adding EV purity (EV particles per μg protein), DNase/RNase/proteinase treatment and EV engineering before *in vivo* therapeutic research. Although current research has not yet standardized these factors, data from all 33 studies in this review suggest that periodontal (dental pulp) cell-derived EVs can function as potential therapeutics to promote periodontal regeneration and impart anti-inflammatory properties. However, investigating the effect of periodontal cell-derived EV on *in vivo* periodontal regeneration models is required to understand their potential therapeutic role in periodontal regeneration.

## Figures and Tables

**Table 1:** Representative advantages and disadvantages of various EV isolation methods.

Method	Time	Advantages	Disadvantage
Ultracentrifuge (100,000×–200,000× *g* for 1–2 h	1.5 h to 10 h	Well-known ‘gold-standard’ method Easy to access Straightforward methodology	Low recovery rate of sEV Time consuming (normally will need 2 steps of UC) Impure sEV with non-EV contamination and aggregates
Floatation-related methods (sucrose gradient centrifugation)	250 min to 1 day	Pure EV population No protein contamination	• Fails to separate large vesicles with similar sedimentation rates
Size exclusion chromatography (SEC)	~30 min (including column washing)	Time-efficient Pure EV product	• sEV and microvesicles cannot be separated
Precipitation based isolation (sodium acetate, PEG, protamine)	Overnight incubation	Low-speed centrifuge (1500 g) to retrieve the sEV sample Straightforward method Many samples can be processed	Low EV recovery Co-precipitation of protein and other molecules Further purification step is required
Immunoaffinity chromatography	~240 min	• Very pure EV subpopulation (i.e., CD9+ EV)	Low EV yield Low scalability
Membrane filtration/Ultrafiltration	~130 min	Small sample volume Simple procedure Higher yield than UC method	• High contamination of non-EV protein

**Table 2:** The isolation, characterization, and function of PDL(S)C-derived EVs.

Reference	Cell Source	EV Isolation	EV Characterization	Key Findings
Xie et al., 2021 [51]	hPDLSCs Donor: Healthy patients aged 18–30 years old, with no system diseases, underwent impacted third molar or orthodontic extractions Passage 3	FBS starvation for 48 h 3000 g for 20 min; 16,500 g for 20 min; 0.2 μm filter; ultracentrifuge at 100,000× *g* for 70 min Centrifuge temperature unclear Exosomes from hPDLSCs before (EX0) and after osteogenic induction for 5 (EX5) and 7 (EX7) days	NTA Flow cytometry TEM WB (CD63 and CD81)	The size of sEV ranged from 20 to 100 nm. 69–557 sEV–circRNAs and 2907–11,581 exosomal lncRNAs were found in EX0, EX5 and EX7 by RNA sequencing. Within hPDLSCs—exosomes, compared with EX0, 3 circRNAs and 2 lncRNAs were upregulated and 39 circRNAs and 5 lncRNAs downregulated in EX5 and EX7. Exosomal circRNAs may function as competing endogenous RNAs through a circRNA–miRNA–mRNA network via TGF-β pathway, MAPK pathway, mTOR pathway and FoxO signaling pathways during hPDLSCs osteogenic differentiation *in vitro*.
Zhang et al., 2020 [52]	hPDLSCs Donors: Periodontal ligaments from premolars of healthy and periodontitis patients Age unclear Passage 2–5	hPDLSCs were cultured with a vesicle-free medium. Centrifuge for 10 min at 500× *g*, 30 min at 16,000× *g*; ultracentrifugation for 70 min at 150,000× *g*	Flow cytometry TEM WB (TSG101 and CD63) SEM	EV size was not mentioned. When co-cultured with TNF-alpha, the angiogenesis of HUVECs was promoted by hPDLSCs exosomes. The angiogenesis of HUVECs was downregulated when the secretion of exosomes was blocked. Inflammation influenced pro- angiogenesis of hPDLSCs via regulating the exosome-mediated transfer of VEGFA targeted by miR-17-5p.
Chiricosta et al., 2020 [53]	hPDLSCs Donor: Five healthy patients from tooth removal for orthodontic purposes Passage 2	Conditioned medium of hPDLSCs at (CM; 10 mL) after 48 h of incubation. 15 min at 3000× *g*; mixed with ExoQuick TC reagent at 4 °C overnight 1500× *g* for 30 min.	No EV characterization performed before RNA sequencing	EVs size was not mentioned The EV derived from hPDLSCs contain several non-coding RNAs, especially the following five miRNAs: miR24-2, miR142, miR296, miR335, and miR490. The target genes of these miRNAs are involved in Ras protein signal transduction and actin/microtubule cytoskeleton organization.
Zhao et al., 2019 [54]	Human periodontal ligament fibroblasts (hPDLFs) cell line MG-63 osteoblast cells line Passages 3–7	P. gingivalis LPS-treated hPDLFs for 4 h and cultured with exosome-depleted FBS/1% PS for another 24 h. 1000× *g* for 10 min and 10,000× *g* for 15 min, 0.22 μm Millipore filter; concentrated CM (3–5 mL) using a 100 kDa ultracentrifuge filter at 5000× *g* in for 30 min; ExoQuick-TC reagent was added and incubated with CM overnight, centrifuge the mixture at 1500× *g* for 30 min	BCA TEM Dynamic light scattering (DLS) WB (CD9, CD63, TSG101)	Exosomes size: 70–100 nm, peaked at 84 nm. Exosome-enriched protein and total exosomal protein levels were higher in the LPS-treated hPDLFs than those in non-LPS-treated hPDLFs. Upregulated IL-6, TNF-α and inhibited expression ALP, collagen-I, RUNX2, and OPG were found in MG-63 OBs after uptaking the exosomes derived from LPS-treated hPDLFs.
Čebatariūn-ienė et al., 2019 [55]	hPDLSCs grew in a bioreactor on gelatin-coated microcarriers Donor: healthy periodontal tissues of two Caucasian females (18 and 21 years old) using explant outgrowth method	Supernatants (SN) from PDLSC in basal medium supplemented with 10% of EV-depleted FCS every 72 h All centrifugation steps were performed at 4 °C 300 g for 10 min, 2000 g for 10 min; 20,000 g for 30 min; then ultracentrifuged at 100,000× *g* for 70 min. The pellets were washed in 40 mL PBS and ultracentrifuged again at 100,000× *g* for 70 min	TEM NTA WB (CD63, MFG-E8)	The size of sEV derived from microcarrier cell cultures of PDLSCs: 112–182 nm. hPDLSCs–sEV suppressed basal and LPS-induced activity of NF-kB in PDLSCs. Combined treatment with EV and anti-TLR4 antibody attenuated the inhibitory effect on the NF-kB activity Uptake of hPDLSCs–sEV in hPDLSC-activated phosphorylation of Akt and GSK3β (Ser 9) indicating that PI3K/Akt signaling pathway may act as a suppressor of NF-kB activity EVs did not significantly affect osteogenic mineralization of hPDLSCs cultures, but EV significantly increased expression of ALP, OPN, BSP, and CP23 gene expression, but downregulated BMP2 expression on the 10 days of osteogenic differentiation.
Pizzicannel-la et al., 2019 [56]	hPDLSCs Donor: Five healthy participants undergoing teeth removal for orthodontic purpose; age unclear Passage 2	The CM from 15 × 10^3^/cm^2^ hPDLSCs was centrifuged at 3000× *g* for 15 min; 2 mL of ExoQuick TC was added to 10 mL of CM; incubated overnight at 4 °C without rotation, centrifuge at 1500× *g* for 30 min, resuspend with 200 μL PBS EVs were coated with branched polyethylenimine (PEI, yielding PEI-EV)	DLS AFM	hPDLSCs–EV: 100–710 nm; engineered PEI–EV: 1050–7700 nm by DLS. *In vitro*: increased expression of osteogenic markers (RUNX2, COL1A1, BMP2/4, VEGFA and VEGFR2) in hPDLSCs cultured in a collagen membrane with EVs and PEI–EVs, as well as increased protein levels of VEGF and VEGFR2. *In vivo*: EV and PEI–EV groups increased VEGF and VEGFR2 protein expression at 6 weeks post transplanting into a rat calvarial defect, as well as enhanced vascular and bone formation. While PEI–EV showed better bone and vascularization than that of the EV group.
Wang et al., 2019 [57]	Human PDL cells (hPDLCs) were prepared from the PDL of fully erupted lower third molar teeth A mouse macrophage-like cell line (J774.1) Human monocyte-like cell line THP-1	Exosome-depleted FBS was prepared using the FBS exosome depletion kit (Norgen, Thorold, ON, Canada) The PureExo R exosome isolation kit (101Bio, Mountain View) was used for exosome isolation. SN was centrifuged at 3000× *g* for 15 min; 2 mL of SN mixed with a solution of PureExo isolation kit and incubated at 4 °C for 30 min, centrifuged at 5000× *g* for 3 min, air-dried, re-suspension in 100 µL PBS; centrifuge for 5 min at 5000× *g*; Processed on a PureExo column, centrifuged at 1000× *g* for 5 min.	TEM WB (CD9) ELISA using PS CaptureTM exosome ELISA kit for CD63	D: 30 and 100 nm (average 50 nm). Cyclic stretch (CS) exposed PDL cells generated 30 times more exosomes compared to non-CS treated normal PDL cells at 24 h CS hPDLC exosomes inhibited IL-1β production in LPS/nigericin-stimulated J774.1 macrophages and the nuclear translocation of NF-kB as well as NF-kB p65 DNA-binding activity in LPS-stimulated macrophages, suggesting that exosomes suppress IL-1β production by inhibiting the NF-kB signaling pathway.
Kang et al., 2018 [58]	hPDLSCs and THP-1 cells lines The passage not mentioned for hPDLSCs	hPDLSCs were treated with 1 μg/mL of LPS for 1 h and CM was collected after 24 h of incubation and filtered using a 0.22 μm filter. CM was centrifuged at 2000× *g* for 10 min at 4 ℃ and filtration through a 0.22 µm filter; ultracentrifuged at 100,000× *g* for 60 min at 4 °C.	NTA WB (CD81 and CD63)	The median sEV particle sizes were 151.3 nm and 146.9 nm for the EVs from control hPDLSCs and LPS-preconditioned hPDLSCs. sEV particle number was significantly decreased in the sEV from LPS-preconditioned PDLSCs compared to those from the control hPDLSCs. sEV from LPS-preconditioned hPDLSCs induced M1 polarization in THP-1 cells, with increased mRNA expression of IL-6 and TNF-α, and TNF-α protein. The M1 polarization was abolished by DNase I treatment of sEV.
Diomede et al., 2018 [59]	hPDLSCs culturing on collagen membranes (Evolution-Evo). Donor: Five participants, either patient for orthodontic purposes or healthy volunteers. Unclear age. Passage 2	After 48 h of incubation, the conditioned medium (CM; 10 mL) was collected from hPDLSCs. CM was centrifuged at 3000× *g* for 15 min; 2 mL ExoQuick TC was added to 10 mL CM; incubated overnight at 4 °C without rotation, centrifuge at 1500× *g* for 30 min EVs were engineered by noncovalently coating EVs with PEI	DLS AFM	DLS analysis showed that hPDLSCs–EV had two populations of vesicles, with an average diameter of 90 ± 20 nm and 1200 ± 400 nm. hPDLSCs–PEI–EV size: 250 ± 50 nm and 3600 ± 500 nm for two populations. Evo enriched with EV and PEI–EV showed high biocompatibility and osteogenic properties both *in vitro* and *in vivo*. PEI–EV promoted the expression of osteogenic genes, such as TGFβ1, MMP8, TUFT1, TFIP11, BMP2, and BMP4 after 6 weeks of *in vitro* osteogenic differentiation in hPDLSCs. PEI–EV group led to an *in vivo* organized extracellular matrix showing mineralization areas and blood-vessel formation, with upregulated BMP2/4 in collagen membrane enriched with PEI–EV and hPDLSCs after 6 weeks in a rat calvarial defect.
Rajan et al., 2016 [60]	hPDLSCs Donor: Five human periodontal ligament biopsies from human premolar teeth of healthy and relapsing-remitting multiple sclerosis (RR-MS) patients.	CM was centrifuged at 3000× *g* for 15 min; 2 mL ExoQuick TC was added to 10 mL CM recovered from hPDLSCs; incubated overnight at 4 °C without rotation, centrifuge at 1500× *g* for 30 min.	Confocal image of CD 63 using fluorescent lipid probes No appropriate characterisation	EV size was not mentioned. After intravenous administration of hPDLSCs–EV into EAE rats, pro-inflammatory cytokines IL-17, IFN-γ, IL-1β, IL-6, and TNF-α were reduced, while the anti-inflammatory cytokine, IL-10, was upregulated. Meanwhile, apoptosis-related STAT1, p53, Caspase 3, and Bax expressions were attenuated. hPDLSCs–EV from MS patients and healthy donors block experimental autoimmune encephalomyelitis (EAE), a mouse model of MS, by inducing anti-inflammatory and immunosuppressive effects in the spinal cord and spleen, and reverse disease progression by restoring tissue integrity via remyelination in the spinal cord.

**Abbreviations:** TGFβ, transforming growth factor β; MAPK, mitogen-activated protein kinase; mTOR, mechanistic target of rapamycin; FoxO, forkhead box protein O; VEGFA, vascular endothelial growth factor A; Ras, Ras GTPase; LPS, lipopolysaccharide; MFG-E8, milk fat globule-EGF factor 8 protein; TLR4, Toll-like receptor 4; PKB/Akt, protein kinase B; GSK-3β, glycogen synthase kinase 3; ALP, alkaline phosphatase; OPN, osteopontin; BSP, bone sialoprotein; CP23, cementum protein 23; BMP2, bone morphogenetic protein 2; RUNX2, runt-related transcription factor 2; COL1A1, alpha-1 type I collagen; VEGFR2, vascular endothelial growth factor receptor 2; IL-1β, interleukin-1 beta; IL-6, interleukin-6; TNF-α, tumor necrosis factor-α; MMP8, matrix metalloproteinase-8; TUFT1, Tuftelin 1; TFIP11, Tuftelin-interacting protein 11; IL-17, interleukin-17; IFN-γ, interferon-γ; IL-10, interleukin-10; STAT1, signal transducer and activator of transcription 1; Bax, Bcl-2-associated X protein.

**Table 3:** The isolation, characterization, and function of EV from hGFs or hGSMCs.

Reference	Cell Source	EV Isolation	EV Characterization	Key Findings
Nakao et al., 2021 [61]	Human gingival mesenchymal stem cells (hGMSCs) Donor details are unclear Passage 4–6	CM was collected after 48 h in FBS-free media and centrifuged at 10,000× *g* for 30 min. hGMSCs exosomes were isolated using MagCapture TM exosome isolation kit PS (FUJIFILM Wako).	TEM NTA WB (CD9, CD63 and CD81)	Mode of sEVs: 109 ± 3.1 nm and 104 ± 1.8 nm for hGMSCs–sEVs and TNF-α pre-treated hGMSCs-derived sEVs. *In vitro*: TNF-α stimulation increased the number of hGMSCs–sEVs and exosomal CD73, as well as induced anti-inflammatory M2 macrophage polarization. The hGMSCs–sEVs–miR-1260b can target Wnt5a-mediated RANKL expression. *In vivo*: in a ligature-induced mice periodontitis model, a local injection of GMSC-derived exosomes significantly reduced periodontal bone resorption.
Yin et al., 2020 [62]	hGFs Donor: Five normal gingival tissues (*n*) and 1 idiopathic gingival fibromatosis (IGF) gingival tissues Passage 4–6	ExoQuick TC (no detailed isolation protocol)	BCA assay TEM FACS (CD63 and CD81)	D: 50–200 nm IGF–GFs–Exo increased cell proliferation of normal hGFs at 24 h and 48 h by MTS assay. The expression of *Ki67*, *PCNA*, *Bcl-2*, and *Bax* were enhanced after 24 h treated with IGF–GFs–Exo. After 48 h, the level of *PCNA*, *Bcl-2* and *Bax* was significantly downregulated, while the expression of Ki67 was not varied significantly.
Zhuang et al., 2020 [63]	hGFs Donor: Fresh human gingiva from donors with wisdom tooth extraction hBMSCs Donor: Fresh human bone marrow from the iliac bone of jaw cysts during the reconstruction of bone defects with hydroxyapatite powder and bone marrow after surgery curettage	Exosome-depleted medium was obtained after 25,000 r.p.m. ultracentrifugation for 90 min. Then, 10 mL of CM was mixed with ExoQuick exosome precipitation solution and refrigerated overnight. CM was centrifuged at 1500 r.p.m. for 30 min at 4 °C and then at 3000 r.p.m. for 5 min.	TEM WB (CD63, CD81 and tubulin)	TEM of EV diameter: 50 to 200 nm. Irradiation-activated hGFs–exosome inhibited osteogenic differentiation of hBMSCs for 7 days, with reduced *ALP*, *COL1* and *RUNX2* gene expression via an exosomal miR-23a/CXCL12 axis.
Silvestro et al., 2020 [64]	hGMSCs Donor: six healthy adult volunteers with no gingival inflammation during teeth removal for orthodontic purpose Passage 2	The conditioned medium (CM; 10 mL) after 48 h of incubation were collected from hGMSCs at passage 2. The CM was centrifuged at 3000× *g* for 15 min; 2 mL ExoQuick TC was added to 10 mL of CM and incubated overnight at 4 °C without rotation; one centrifugation step was performed at 1500× *g* for 30 min to sediment the EVs	No EV characterization	The size of EV was not mentioned. RNA sequencing analysis showed that 15,380 genes were identified in GMSCs–EVs. There were 1067, 886, 808, 768, 562, and 541 protein-coding genes for hydrolase, enzyme modulators, the transcription factor, transferase, the receptor and the transporter, respectively. There were 1155 non-coding RNA genes for anti-sense RNAs, lncRNAs and miRNAs (miR1302 family, miR451 family, miR24 family, miR219 family and miR194 family). hGMSCs–EV also contain mRNAs for proteins of the interleukins, TGF-, BMPs, GDFs, Wnt, VEGF, FGF, and neurotrophins are critical for basic or neuronal, bone or vascular development.
Coccè et al., 2019 [65]	Human MSCs were isolated from gingival papilla (named GinPaMSCs) Donor details are unclear	GinPaMSCs cultured with FBS-free media for 72 h cultures before EV collection. CM was collected at 24 and 48 h of incubation and centrifuged on a 100 kDa filter device at 5000× *g* for 15 min. The two fractions (i.e., EV: F > 100 kDa; free PTX: F < 100 kDa)	DLS NTA TEM	TEM of exosomes: 50 to 500 nm. DLS of EV: 200–300 nm. NTA detected the following 3 different EV populations: 135 nm, 200–300 nm, and 435 nm. PTX was presented in PTX-treated GinPaMSCs-secreted EVs. *GinPaMSCs*–EV/PTX have anti-cancer activity in human pancreatic carcinoma and squamous carcinoma cells.
Mao et al., 2019 [66]	hGMSCs: gingival tissues were obtained from five healthy human subjects aged from 20-to-40 years, who underwent routine dental procedures A rat Schwann cell (SC) line RT4-D6P2T hGMSCs: passage < 6 SCs passage < 4	hGMSCs cultured in media with 1% exosome-depleted FBS (System Biosciences, SBI) for 48 h to collect CM. The CM was centrifuged at 1000× *g* for 30 min; 20 mL of culture media was filtered a Vivaspin 20 ultrafiltration device (100 kDa) and centrifuged at 3000× g for 60 min to get 1 mL of the concentrated medium; mixed with 0.2 mL ExoQuick-TC exosome precipitation solution (5:1) and incubated overnight at 4 °C; centrifuged at 1500× *g* for 30 min at 4 °C	BCA assay NTA WB (CD 63 and CD9)	GMSC-derived sEV had a mean size of 103.8 ± 2.1 nm by NTA. *In vivo* studies mimicking clinical nerve repair showed that hGMSCs-derived sEV promoted functional recovery, axonal repair and regeneration of crush-injured mice sciatic nerves. *In vitro*: GMSC-derived EVs promoted proliferation and migration of Schwann cells, with upregulated c-JUN, Notch1, GFAP (glial fibrillary acidic protein), and SRY (sex-determining region Y)-box 2 (SOX2).
Diomede et al., 2018 [67]	hGMSCs seeded on 3D poly (lactide) (PLA) scaffolds Donor: gingival tissue biopsies were obtained from healthy adult volunteers with no gingival inflammation	After 48 h of incubation, EVs were collected from hGMSCs at passage 2. centrifuged at 3000× *g* for 15 min; 2 mL ExoQuick TC was added to 10 mL CM and incubated overnight at 4 °C; 1500× *g* for 30 min to sediment the EVs EVs were engineered with PEI (MW 25,000).	DLS AFM WB (CD9, CD63, CD81, and TSG101)	DLS of hGMSCs–EVs: 93 ± 24 nm and 1200 ± 400 nm; engineered hGMSCs–PEI–EV: 250 ± 50 nm and 3600 ± 500 nm. EV and PEI–EV increased calcium deposits after 6 weeks of osteogenic differentiation in hGMSCs, with increased RUNX2 and BMP2/4 gene and protein expression. RNA sequencing of the transcriptome of hGMSCs, EV and PEI–EV revealed that 31 genes were differentially expressed between groups. GO analysis showed that these 31 genes involved in “regulation of ossification” and “ossification” were upregulated in the PEI–EV group compared to hGMSCs group through the TGF-β signaling. *In vivo* results showed that PEI–EV with/without hGMSCs in PLA scaffolds enhanced bone and blood vessels formation in a rat cortical calvaria defect by histology and microCT.

**Abbreviation:** MTS, 3-(4,5-dimethylthiazol-2-yl)-5-(3-carboxymethoxyphenyl)-2-(4-sulfophenyl)-2H-tetrazolium; Ki-67, antigen KI-67/marker of proliferation Ki-67; PCNA, proliferating cell nuclear antigen; CXCL12, C-X-C motif chemokine 12/ stromal cell-derived factor 1; FGF, fibroblast growth factor; GDNF, glial cell-derived neurotrophic factor; PTX, paclitaxel; c-JUN, jun proto-oncogene/AP-1 transcription factor subunit; Notch1, Notch homolog 1(translocation-associated).

**Table 4:** The isolation, characterization, and function of hDPSCs–EV.

Reference	Cell Source	EV Isolation	EV Characterization	Key Findings
Faruqu et al., 2020 [68]	Human dental pulp pluripotent-like stem cells (hDPPSCs) from healthy human third molars extracted for orthodontic and prophylactic reasons Umbilical cord-derived mesenchymal stem cells The passage is not mentioned	Exosome-depleted FBS was obtained after ultracentrifugation at 100,000× *g* for 18 h at 4 °C. CM was filtered through 0.22 μm filter, mixed with sucrose solution with 25% *w*/*w* in deuterium oxide (D2O) and ultracentrifugation at 100,000× *g* for 1.5 h at 4 °C and another 100,000× *g* for 1.5 h at 4 °C.	NTA Dot blot (CD9, CD63, alix, TSG101, and calnexin)	sEV from hDPPSCs spheroids culturing in KnockOut™ serum replacement (KO-medium) at both day 1–12 and day 13–24 samples were detected similar particle sizes (168.7 ± 7.2 nm and 156 ± 7.6 nm).
Zhou et al., 2020 [69]	hDPSCs Donor: 5 healthy donors (male: 2; female: 3; age: 24~41 years) and periodontitis teeth (n = 6), named H-DPSCs and P-DPSCs Passage 3~5 H-DPSCs and P-DPSCs-derived EVs are named H-EV and P-EV.	At 90% confluence, hDPSCs were washed three times with PBS prior to culturing with serum-free media for 48 h. CM was centrifuged at 300× *g* for 10 min; 2000× *g* for 10 min; centrifuged at 10,000× *g* for 30 min; ultracentrifuged at 100,000× *g* for 70 min; washed with PBS at 100,000× *g* for 70 min	TEM NTA WB (alix, HSP70, CD9, CD63 and CD81) BCA assay	Both H-EV and P-EV sizes ranged from 30 to 200 nm. hPDSCs–sEV from periodontitis patients promoted endothelial cells proliferation and angiogenesis with higher expression levels of VEGF and AngII genes/proteins compared with hPDSCs–sEV from healthy patients. *In vivo*: The vascularization of mouse skin defects and wound healing were promoted by both H-EV and P-EV, while the treatment with the latter brought a faster repairing process, as well as the formation of fresh vessels.
Ivica et al., 2020 [70]	hDPSCs from healthy third molars (n = 3, 16 to 25 years old) were extracted Human bone marrow-derived mesenchymal stem cells (HBMMSCs). Passage 3–6	Exosome-free medium (Invitrogen) was used for hDPSCs culture for 48 h before CM collection. CM centrifuged at 300× *g* for 10 min; 2000× *g* for 30 min; total exosome isolation agent (Invitrogen) was added; the mixture was centrifuged at 10,000× *g* for 30 min	TEM WB (CD9, and Grp94)	hDPSC–sEV ranged from 45 to 156 nm by TEM, with a mean size of 90 nm. hDPSC–sEV encapsulated in fibrin gel promoted hBMMSCs migration and proliferation.
Xie et al., 2020 [71]	hDPSCs Donor: one healthy patient aged 20 years old, free of periodontal or endodontic problems.	Exosomes secreted by hDPSCs during the starvation of 48 h without FBS, marked as EX0. Osteogenic-induced DPSC-derived exosomes (OI-DPSC-Ex) after culturing with osteogenic media with 15% exosome-free FBS. Exosomes secreted by these osteogenic-induced DPSCs at days 5 and 7 were extracted and marked as EX5 and EX7. CM was centrifugated at 3000× *g* for 20 min; centrifuged at 16,500× *g* for 20 min; filtered with a 0.2 micron filter to collect the filtrate; ultracentrifuged at 100,000× *g* for 70 min Passage 2	TEM NTA Flow cytometry (CD63 and CD81)	TEM and NTA detected that exosomes size range from 20 to 120 nm. The OI-DPSC-Ex induced the osteogenic differentiation of recipient parent hDPSCs via exosomal circPAR1 binding with hsa-miR-31.
Shen et al., 2020 [72]	hDPSCs Donor: from exfoliated teeth of healthy donors Passage not mentioned	80% confluent hDPSCs cultured with medium supplemented with 10% exosome-free FBS (centrifuged at 120,000× *g* for 18 h) for 3 days. CM was centrifuged at 300× *g* for 10 min; centrifuged at 16, 500× *g* for 20 min; ultracentrifuged at 120,000× *g* for 2.5 h at 4 °C DPSC-Exo encapsulated in chitosan hydrogel (CS) was named DPSC-Exo/CS	TEM NTA WB (CD9, HSP70 and TSG 101) Flow cytometry (CD 63 and CD 81)	NTA of EV: 50 to 400 nm, peaked at 155.4 nm. In an *in vivo* periodontitis mice model, DPSC-Exo/CS promoted the regeneration of alveolar bone and periodontal epithelium in the periodontitis mice model after 10 days. DPSC-Exo/CS had anti-inflammatory effects and facilitated the immune response by switching macrophages from a pro-inflammatory phenotype to an anti-inflammatory phenotype at both *in vitro* and *in vivo* mice with periodontitis via hDPSCs-derived exosomal miR-1246.
Li et al., 2019 [73]	hDPSCs Donor: healthy patients (aged 19–28 years old, n = 12) impacted third molars LPS pretreated overnight in hDPSCs. EV from LPS-treated cells were named LPS-exo Passage 3–5	hDPSCs at 70–80% confluence cultured for 48 h in media exosome-depleted FBS (Systembio). CM was centrifuged at 500× *g* for 10 min at 4 °C, centrifuged at 2000× *g* for 10 min; centrifuged at 10,000× *g* for 1 h at 4 °C and filtrated through a 0.22 μm filter; ultracentrifuged at 100,000× *g* for 2 h; ultracentrifuged at 100,000× *g* at 4 °C for 70 min	BCA assay WB (alix, CD9, CD63 and GM130) TEM NTA	EV: 80–400 nm, peaked at 116 nm. LPS-treated cells generated more exosomes particles. Both exo and LPS-exo facilitated the production of dentin sialoprotein and mineralization of Schwann cells (SCs). LPS-exo had a higher promoted proliferation, migration and odontoblast differentiation of Schwann cells (SCs) compared to Exo only, with increased DSPP, DMP1, OCN, and RUNX2 gene expression after 14 days.
Ji et al., 2019 [74]	hPDSCs were isolated from healthy dental pulp tissues and (n = 8) from the caries-free teeth that need to be extracted due to orthodontics hBMMSCs were isolated from bone marrow aspirates of healthy people (n = 8).	At 80% confluence, the medium was replaced with a serum-free medium for 48 h before CM collection. The CM was centrifuged at 300× *g* for 10 min, centrifuged at 16,500× *g* for 30 min at 4 °C; passed through a 0.2 μm; ultracentrifuged at 4 °C at 100,000× *g* for 70 min; ultracentrifuged again at 4 °C at 100,000× *g* for 70 min	TEM NTA BCA WB (CD9 and CD63)	NTA of EV: 30–250 nm, mean size of 135 nm. hDPSCs–EV suppressed the differentiation of CD4+ T cells into T helper 17 cells (Th17), but stimulated the polarization of CD4+ T cells into regulatory T cells (Treg). hDPSCs–EV and hBMMSCs–EV inhibited the secretions of pro-inflammatory factors (IL-17 and TNF-α) and increased anti-inflammatory factors (IL-10 and TGF-β) in CD4+ T cells after 72 h of treatment. Both hDPSCs–EV and hBMMSCs–EV inhibited proliferation, but increase the apoptosis of CD4+ T cells.
Hu et al., 2019 [75]	hDPSCs Donor: healthy pulp tissues isolated from caries-free teeth of patients (5 females, age 24–35 years; 5 males, age 22–36 years) undergoing extraction of fully erupted third molars Passage 3–7	Exosomes from hDPSCs in either growth (UN-Exo) or odontogenic differentiation media (OD-Exo) for 10 days Cells were washed in serum-free PBS and cultured for 48 h in serum-free media. The Exo-spin (Cell Guidance) exosome isolation reagent (no detailed description)	WB (CD9 and CD63) TEM	UN-Exo and OD-Exo range from 30 to 150 nm in diameter by TEM. OD-Exo promoted the odontogenic differentiation in hDPSCs with increased *RUNX2*, *DMP-1*, *DSP* and *ALP* gene expression. RNA sequencing analysis showed that 28 microRNAs significantly changed in OD-Exo isolated under odontogenic conditions, of which 7 miRNAs increased and 21 miRNAs decreased. The qRT-PCR analysis showed that miR-5100 and miR-1260a levels in OD-Exo increased, while miR-210-3p and miR-10b-5p decreased, which were consistent with the miRNA sequencing. GO analysis showed that the differentially expressed miRNAs are associated with the TGFβ1 pathway. OD-exo activated the TGFβ1 pathway by upregulating TGFβ1, TGFR1, p-Smad2/3, and Smad4 in DPSCs, compared to the control group and UN-Exotreated group. Compared with UN-Exo, miR-27a-5p was expressed 11 times higher in OD-Exo. The luciferase reporter assay demonstrated that miR-27a-5p can target the 3′-UTR of LTBP1 directly. This suggests that exosomal miRNAs promoted odontogenic differentiation via the TGFβ1/smads signaling pathway by downregulating LTBP1.
Huang et al., 2016 [76]	hDPSCs and primary hMSCs are not clearly detailed. Both hDPSCs and hMSCs used were passage <4	Exosomes were isolated from hDPSCs in either growth (DPSC-Exo) or odontogenic differentiation media (DPSC-OD-Exo) for 4 weeks. Cells were washed in serum-free media and cultured for 24 h in serum-free media before CM collection. ExoQuick-TC exosome isolation reagent (non-detailed description)	WB (CD63 and CD9) TEM	Size of EV was not demonstrated. The endocytosis of exosomes was dose-dependent and manner-saturable for both hMSCs and hDPSCs *in vitro*, which initiated the MAPK pathway through the caveolar endocytic mechanism. hDPSCs–EV can bind to fibronectin and type I collagen via an exosomal integrin-mediated process. DPSC-Exo and DPSC-OD-Exo induce increased expression of odontogenic marker genes in DPSCs in 3D culture within type I collagen hydrogels. DPSCs-Exo and DPSC-OD-Exo in a collagen membrane on a filled root canal spaces of human tooth root slices were implanted subcutaneously in athymic nude mice for 2 weeks. DPSC-Exo and DPSC-OD-Exo triggered increased expression of odontogenic differentiation marker proteins DMP1 and DPP, and only the DPSC-OD-Exo group improved active blood vessels formation and endothelial cell marker von Willebrand factor (vWF). DPSC-Exo and DPSC-OD-Exo in hMSCs after 48 h of *in vitro* treatment increased in the expression levels of several growth factors and ECM proteins along with the transcription factor Runx2.

**Abbreviations**: circPAR1, circular Prader–Willi/Angelman region-1; DMP-1, dentin matrix acidic phosphoprotein 1; DSPP, dentin phosphophoryn; TGFR1, transforming growth factor beta receptor I; p-Smad2/3, mothers against decapentaplegic homolog 2/3 or SMAD family member 2/3; Smad4, SMAD family member 4/mothers against decapentaplegic homolog 4; UTR, untranslated region; LTBP1, latent TGF-beta binding protein.

**Table 5:** The isolation, characterization, and function of sEVs from SHED, SCAP and DFCs.

Reference	Cell Source	EV Isolation	EV Characterization	Key Findings
Liu et al., 2021 [77]	Stem cells from apical papilla (SCAP) Donor: healthy third molars with immature roots from healthy donors aged 12 to 15 years Passage number is unclear	SCAP cells were cultured in exosome-free medium for 48 h and CM was centrifuged at 4 °C in an ultracentrifuge at the following three different speeds: 3000× *g* for 20 min, 20,000× *g* for 30 min, and 120,000× *g* for 2 h Ultracentrifugation method	TEM NTA WB (CD9, CD63, and Alix)	sEVs mode: 120.1 nm; mean: 139.2 ± 62.5 nm *In vivo*: SCAP–sEVs promoted vascularization to accelerate tissue regeneration of the palatal gingiva via Cdc42-mediated vascularization in a mouse gingival wound healing model. *In vitro*: SCAP–sEVs enhanced the cell migration and angiogenic capacity of HUVECs via Cdc42-mediated cytoskeletal reorganization.
Zhuang et al., 2021 [78]	SCAP Donor: Human impacted third molar with immature roots were collected from healthy patient (12–15 years old) Passage number is unclear	SCAP cells at 60–80% confluence were cultured with serum-free media for 48 h before CM collection. The CM was centrifuged sequentially at 4 °C: 3000× *g* for 20 min, 20,000× *g* for 30 min, and 120,000× *g* for 2 h. Ultracentrifugation	TEM NTA BCA WB (CD9, and Alix)	sEVs peaked at 120.6 nm. SCAP-Exo promoted mouse BMMSC-based dentine-pulp complex regeneration *in vivo* and *in vitro* dentinogenesis of BMMSCs.
Wang et al., 2020 [79]	Stem cells from human-exfoliated deciduous teeth (SHED) hPDLCs Donor: age is unclear; pulp tissue from non-carious primary teeth extracted from children for orthodontic reasons Passage: 3–4	SHED and hPDLCs cells were cultured in 15% and 10% exosome-free media, respectively. The CM was centrifuged at 300× *g* for 10 min, 2000× *g* for 10 min, and 20,000× *g* for 30 min and 100,000× *g* for 70 min Ultracentrifugation	TEM BCA NTA WB (CD9, CD63, TSG101 and Calnexin)	DLS revealed that the SHED–sEVs diameter ranges from 40 to 140 nm. SHED–sEVs promote *in vitro* osteogenic differentiation in PDLCs via Wnt/β-catenin and BMP/Smad signaling pathways.
Wei et al., 2020 [80]	SHED were purchased Mouse bone marrow stromal cells (mBMSCs) were isolated from femur and tibia bone marrow of CD-1 mice (9–10 months old). Passage: 4–7 for SHED–sEVs	At 70% confluence, SHED cells were cultured in serum-free media for 24 h. The CM was collected and centrifuged at 300× *g* for 10 min, 2000× *g* for 10 min, 10,000× *g* for 60 min before a 0.22 μm filter. 100,000× *g* for 70 min twice to pellet sEVs. Ultracentrifugation	TEM WB (CD63)	Diameter: ~100nm *In vivo*: local injection of SHED–sEVs rescued ligature-induced periodontitis bone loss in mice. *In vitro*: SHED–sEVs promoted cell proliferation, osteogenesis and reduced adipogenesis and inflammatory cytokines secretion in mBMSCs.
Luo et al., 2019 [81]	SHED was purchased from a cell bank and cells were from nine normal human deciduous incisors collected from 5- to 8-year-old individuals. Chondrocytes were isolated from cartilage tissues of five patients with condylar fracture Passage 4 for both SHED and chondrocytes	SHED cells were confluent before culturing in exosome-free media for 48 h. CM was collected and centrifuged at 4 °C: 300× *g* for 10 min, 2000× *g* for 10 min, 20,000× *g* for 30 min and 100,000× *g* for 70 min Ultracentrifugation	TEM NTA WB (CD9, CD63 and TSG101)	The sizes of SHED–sEVs range from 30 to 100 nm. SHED–sEVs inhibited pro-inflammatory cytokines expression in chondrocytes *in vitro* via an exosomal miR-100-5p and mammalian target of rapamycin (mTOR) pathway.
Wu et al., 2019 [82]	SHED and HUVECs cells: commercial cells rBMSCs: femoral bones of SD rats Passage: 3–6 (SHED)	Exosome-depleted FBS was obtained after ultracentrifuging at 100,000× *g* for 12 h. CM collection was not clear. The CM was centrifuged at 300× *g* for 10 min, and 2000× *g* for 15 min, 10,000× *g* for 30 min and concentrated using ultrafiltration, followed by centrifuging at 100,000× *g* for 1 h. Ultracentrifugation	TEM NTA BCA WB (CD81, CD9 and TSG101)	D: 50 to 200 nm, with two peaks, at 101 and 144 nm. *In vitro*: SHED-–sEVs promoted proliferation, migration and angiogenesis in HUVECs and osteogenic differentiation in rBMSCs via adenosine monophosphate-activated protein kinase (AMPK) pathway. *In vivo*: SHED–sEVs promoted neovascularization and new bone formation in a periodontal bone defect rat model.
Shi et al., 2020 [83]	Dental follicle cells (DFCs) Donor: dental follicle tissue obtained from the immature third molars was selected in young patients (age 13−19 years). hPDLCs from chronic periodontitis (age 40−55 years). Passage: 5 (for DFCs)	At 80% confluence, DFCs were treated with/without LPS for 24 h prior to culturing in serum-free media for 48 h. The CM was centrifuged at 2000× *g* for 30 min and filtered by a 0.22 μm filter. Then the supernatant was ultrafiltered using 100 KD ultrafiltration at 5000× *g* or 30 min. Then total exosome isolation reagent was added to the concentrated solution and put into a 4 °C refrigerator overnight and centrifuged at 10,000× *g* for 1 h. Ultracentrifuge	TEM NTA WB (CD63 and TSG101)	The diameter of DFCs–sEVs was peaked at 120nm. LPS precondition increased the secretion of sEV from DFCs. *In vitro*: LPS–DFCs–sEVs promoted proliferation, migration and osteogenic differentiation in periodontitis derived hPDLCS. *In vivo*: LPS–DFCs–sEVs enhanced orientated periodontal ligament formation, periodontal bone formation, as well as reduced TRAP-positive osteoclasts cells and RANKL/OPG expression.

**Abbreviations:** Cdc42, cell division control protein 42 homolog; RANKL/OPG, receptor activator of nuclear factor kappa-B ligand/osteoprotegerin.

## Data Availability

Not applicable.
